# The cost of child health inequalities in Aotearoa New Zealand: a preliminary scoping study

**DOI:** 10.1186/1471-2458-12-384

**Published:** 2012-05-28

**Authors:** Clair Mills, Papaarangi Reid, Rhema Vaithianathan

**Affiliations:** 1 Te Kupenga Hauora Māori, Faculty of Medical and Health Sciences, University of Auckland, Auckland, New Zealand; 2 Department of Economics, The University of Auckland Business School, Auckland, New Zealand; 3 Northland District Health Board, Whangarei, New Zealand

## Abstract

**Background:**

Health inequalities have been extensively documented, internationally and in New Zealand. The cost of reducing health inequities is often perceived as high; however, recent international studies suggest the cost of “doing nothing” is itself significant. This study aimed to develop a preliminary estimate of the economic cost of health inequities between Māori (indigenous) and non-Māori children in New Zealand.

**Methods:**

Standard quantitative epidemiological methods and “cost of illness” methodology were employed, within a Kaupapa Māori theoretical framework. Data were obtained from national data collections held by the New Zealand Health Information Service and other health sector agencies.

**Results:**

Preliminary estimates suggest child health inequities between Māori and non-Māori in New Zealand are cost-saving to the health sector. However the societal costs are significant. A conservative “base case” scenario estimate is over $NZ62 million per year, while alternative costing methods yield larger costs of nearly $NZ200 million per annum. The total cost estimate is highly sensitive to the costing method used and Value of Statistical Life applied, as the cost of potentially avoidable deaths of Māori children is the major contributor to this estimate.

**Conclusions:**

This preliminary study suggests that health sector spending is skewed towards non-Māori children despite evidence of greater Māori need. Persistent child health inequities result in significant societal economic costs. Eliminating child health inequities, particularly in primary care access, could result in significant economic benefits for New Zealand. However, there are conceptual, ethical and methodological challenges in estimating the economic cost of child health inequities. Re-thinking of traditional economic frameworks and development of more appropriate methodologies is required.

## Background

Health inequalities have been extensively documented, internationally and in New Zealand [1-4]. The cost of reducing health inequities (defined here as those inequalities in health between population groups that are unnecessary, preventable, and amenable to policy intervention [4-6]) is often perceived as high. However, recent international studies suggest the cost of “doing nothing” is itself significant. Studies from Europe and North America estimate the economic costs of health inequities in adults as 2–4.4% of Gross Domestic Product, and 15–20% of health sector expenditure [7-13].

Health inequities between indigenous Māori children and non-Māori children in New Zealand remain significant and persistent, particularly in relation to infectious diseases, injury and infant and child mortality rates. For example, Māori infants have rates of Sudden Unexpected Death in Infancy (SUDI) over six times non-Māori infants, are nearly three times more likely than non-Māori to be hospitalised with bronchiolitis, and as children have much higher rates of rheumatic fever and bronchiectasis than non-Māori [3]. This scoping study aimed to carry out a preliminary analysis of the cost of health inequities between Māori and non-Māori children in New Zealand.

## Methods

### Kaupapa Māori methodology

This study is positioned within a Kaupapa Māori theoretical framework, a research approach that is critically mindful of how research processes may contribute to the marginalisation of indigenous peoples [14,15]. Kaupapa Māori methodology is driven by a Māori world view and recognises the complexity of Māori historical and contemporary realities. Understanding the current health inequities experienced by Māori children and young people requires an understanding of New Zealand’s colonial history and the erosion of the social, economic, political, and cultural base for Māori whānau and iwi (families and kinship groups/tribes), from the early 1800s onwards [3]. Kaupapa Māori methodology privileges the indigenous Māori “voice”, validating and legitimising Māori language, knowledge, culture and values. It acknowledges and challenges the power dynamics that have created and maintain the unequal position of Māori in New Zealand society, including the role played by the socio-economic determinants of health and the health system in limiting Māori health outcomes [15].

In this study, quantitative epidemiological and economic costing methods are used as tools, with questions and findings developed and interpreted within a Kaupapa Māori framework. The analysis reflects this understanding by placing Māori children as the group “at the centre” of this analysis rather than at the margins.

### Measures of child health inequity

The focus of this study is on inequities; therefore our analysis is based on the deaths and hospitalisations that are categorised as “potentially avoidable”, using New Zealand Ministry of Health and District Health Board (DHB) definitions [16-18]. This includes illness and injury preventable through primary care intervention, injury prevention or health promotion approaches. The International Statistical Classification of Diseases and Related Health Problems, Tenth Revision, Australian Modification (ICD-10-AM) diagnostic coding was used for all admissions and deaths [19]. We assigned children to Māori and non-Māori groups using Ministry of Health protocols for prioritised ethnicity; that is, anyone who identified as Māori, either alone or as one of multiple ethnic groups, was included in the Māori group [20].

Datasets were obtained with permission from Statistics New Zealand (SNZ) and the New Zealand Health Information Service (NZHIS). We calculated the number and crude rates of death from avoidable causes by ethnicity and age group (age groups: 0–1 month, 1 month < 1year, 1- < 5years, 5- < 15years) using SNZ death registration data coded by NZHIS for 2003–2007. Live births from 2003–2007 (SNZ birth registration data, excluding stillbirths, coded by NZHIS) were used as the denominator to estimate neonatal (<28days) and post-neonatal (defined as 28days- < 1year) infant mortality and hospital admission rates [unpublished datasets, SNZ and NZHIS]. For mortality rates in children over one year, population estimates from New Zealand Census data were used to estimate Māori and non-Māori rates [21,22].

Avoidable deaths were assigned by their primary ICD-10AM code into ICD-10AM chapter groupings. The number of avoidable deaths that would have occurred had Māori had the same rate as non-Māori in each age band was computed, with the difference between the actual number of deaths and the estimated deaths representing excess avoidable deaths. The resulting number of years of life lost (YLL) by Māori children was computed, assuming that all would have lived to the 2005 life expectancy of their non-Māori same-sex counterparts [23].

We obtained the number and crude rates of hospitalisations from potentially avoidable causes by ethnicity and age group (Māori, non-Māori; age groups: 0- < 1year, 1- < 5years, 5- < 15years) from hospitalisation data (National Minimum Dataset, NZHIS) for the same period, 2003–2007. The number of avoidable hospitalisations that would have occurred if Māori had the same rate as non-Māori in each age band and diagnostic grouping (ICD-10AM chapter) was computed, with the difference between the actual number of hospitalisations and the estimated hospitalisations representing excess avoidable hospitalisations.

We assumed that injuries were as avoidable for Māori as for non-Māori children, thus that any difference in injury rates was inequitable, and that the incidence of injury and primary care-responsive illness was at least as high in Māori as non-Māori children [24,25]. Primary care utilisation rates were estimated based on Primary Health Organisation (PHO) service utilisation data for visits for enrolled populations during routine hours for the years 2007–8 [unpublished datasets, used with permission of District Health Boards NZ]. Total utilisation rates were compared, as the quality of diagnostic coding in primary care precluded sub-group analysis. The number and rate of general practice (first point of contact personal and family primary medical care services) and primary care nursing visits were calculated for Māori and non-Māori children by age group (0- < 5years, 5- < 15years). The number of consultations that would have occurred if Māori had the same rate as non-Māori were computed by age group, with the difference between the actual number of visits and the estimated number representing “excess” or “under-utilisation.”

Similar methods were applied to Accident Compensation Corporation (ACC) injury claims, pharmaceutical and laboratory claims and outpatient datasets, estimating excess or under-utilisation for total claims/visits. Laboratory and pharmaceutical data have only recently been linked to NHI numbers (and thus able to be disaggregated by ethnicity), so we used the most recent years available (2006–8), as NZHIS advised NHI linking was then over 95% complete [personal communication, NZHIS].

### Economic costing methods

We reviewed the available literature on costing health inequities and assessed relevant economic methods. This review suggests that there are significant unresolved challenges in valuing child health and disability that continue to be debated in the literature. Many of the assumptions of neoclassical economics do not hold for children and are indifferent to concepts of equity [26-32]. Monetising the value of a child’s life and discounting future benefits (used by economists to reflect the assumption that future benefits are less valued than benefits enjoyed in the present) remain contentious in the health sector and do not fit easily within a kaupapa Māori framework. Re-interpreting cost benefit analysis theory within such a framework is an interesting direction for future kaupapa Māori research. In the absence of this, we chose to use “cost of illness” methods [33-37] in this preliminary study, with a limited prevalence-based perspective. This restricts estimates to a range of publicly funded health sector costs, the cost of premature mortality, “out of pocket” care-giver expenditure on primary care, and loss of days of work for care-givers, due to excess avoidable hospital admissions of their children. We estimated an annual cost based on the total costs of the “inequity excess” for the period 2003–7.

### Costs of health care

Costing data for hospital admissions was based on the National Pricing Project (NPP) 2008–9 dataset [unpublished data, Ministry of Health]. The direct hospital cost savings (or extra costs) if Māori children had avoidable admission rates equal to non-Māori children was computed using the weighted median Diagnostic Related Group (DRG) costs for each ICD-10AM category and age group. ACC injury claims cost data, Pharmaceutical Management Agency of New Zealand (PHARMAC) pharmaceutical claims and NZHIS laboratory services claims for 2007–8 were used to cost those services. Māori:non-Māori differences in the use of ACC, laboratory and pharmaceutical services were costed using the weighted median costs of non-Māori claims in each age group. Paediatric outpatient visits (including mental health) were assigned the Weighted Inlier Equivalent Separations (WIES) inter-district flow cost (2008–9) for a paediatric medical outpatient visit of $343.23.

We estimated the difference in primary care costs using the “notional” Ministry of Health general medical subsidy amount per visit and assumed a nursing visit total cost of $30 [38,39].

### “Out of pocket” costs in primary care and costs of parental days of work lost

For primary care, we conservatively assumed the median “out of pocket” expenditure for children under six in 2007–2008 as zero, that children aged six and over were all subsidised $15 for general practitioner (family doctor) and nursing visits, and that caregivers “out of pocket” payments represented the difference between that and the notional visit cost [38-40]. A conservative estimate of the cost of work days lost (measured as wages) was based on loss of two days work (the weighted median length of stay in this study) for each “excess” admission, for one parent at the 2007–2008 gross median wage ($NZ35,000) [41].

### Costs of years of life lost

We used non-Māori male and female life expectancy from SNZ Life Tables 2005–7 [23] to derive the excess avoidable “Years of Life Lost” (YLL). Various discounting rates were then applied to test the impact of different time preferences, using discount rates applied in recent New Zealand cost of illness studies [33-36].

Two methods were used to assign costs to YLL. Firstly, we assumed a “base case” scenario of a mean life expectancy at birth of 78 years, a 3% discount rate, and estimated the total cost of YLL by dividing the “base case” Value of a Statistical Life (VoSL) of $NZ3.352million [35,36] by the Statistical Lives lost (“Method 1”). We assumed that the VoSL included lost production costs.

We also computed costs applying standard economic methods used by O’Dea & Tucker and others [34,35] (“Method 2”) to allow comparability with other New Zealand cost of illness studies. Using this methodology, the VoSL has the same value for both genders, but given life expectancy is gender-dependent, the value of a “year of life lost” varies depending on age and gender of the avoidable deaths. The annualised value of the VoSL is calculated using the equation VosL = Sum [VoSLY/(1 + d)^L^, where d is the discount rate and L the life expectancy. Therefore the VoSLY = VoSL*d/[1-(1 + d)^−L^, where d = the discount rate, and L is life expectancy.

## Results

Table 1 summarises the results for avoidable hospitalisations and healthcare costs of differential utilisation by Māori and non-Māori children. The estimate of the health sector costs of inequity in childhood illness and injuries is a cost saving to the health sector of $24,737,408 per annum.

**Table 1:** Māori: non-Māori Rate Ratios and Estimates of the Cost to the Health Sector of Inequity in Illness and Injury ($NZ)

	**Māori: non-Māori Rate Ratio**	**If Māori rates equaled non-Māori rates:**	**Cost[-savings]/year (0% discount rate)**
**Avoidable hospital admissions (2003–7)**	**1.27** (95% CI 1.26–1.28)	3075 fewer Māori admissions/year	**$ 5,671,057**
**Outpatient consultations (2006–2008)**	**0.864** (95% CI 0.862–0.867)	23,373 more consultations/year	**[−]$ 8,022,315**
**Mental health consultations (20007–8)**	**0.719** (95% CI 0.714–0.723)	5740 more consultations/year	**[−]$ 1,970,140**
**ACC claims (2003–7)**	**0.680** (95% CI 0.678–0.683)	26,442 more claims/year	**[−]$ 1,499,765**
**Primary care (2007–2008)**	**0.919** (95%CI 0.916–0.921) GP visits	40,041 more GP consultations/year	**[−]$ 1,079,906**
	**1.677** (95%CI 1.665–1.689) nursing visits	17,194 fewer nursing consultations	**$ 400,803**
**Pharmaceutical claims (2007–8)**	**0.849**	198,108 more claims/year	**[−]$ 10,447,348**
**Laboratory claims (2006–8)**	**0.454** (95%CI 0.452–0.455)	101,922 more claims/year	**[−]$ 7,789, 795**
**SUB TOTAL HEALTH SECTOR COSTS/[SAVINGS] PER ANNUM**			**[−]$ 24,737,408**

### Inequity in potentially avoidable hospital admissions

There were a total of 871,094 hospital admissions for children <15 years in the period 2003–2007. The crude rate ratio for total Māori:non-Māori admissions was 0.98 (95% CI 0.975–0.984). 36% of hospitalisations of children in this age group were classified as “potentially avoidable”. Māori:non-Māori rate ratios for potentially avoidable hospitalisation rates were significantly greater than one in all age groups. Respiratory diseases made the greatest contribution to the “excess”, with diseases of the ear, digestive system and skin, and injuries also being important. In children aged 5–15years the highest rate ratio was for circulatory diseases (including rheumatic heart disease), where the Māori:non-Māori rate ratio was 3.33 (95% CI 2.93–3.79). Overall, summing the “excess” avoidable hospitalisations, there were a total of 15,376 “excess” Māori avoidable admissions during 2003–7 or 3,075 each year.

### Inequity in hospital general outpatient and mental health outpatient consultations (2006–2008)

Hospital outpatient utilisation rates for Māori children were consistently lower than for non-Māori children for all age groups in the period July 2006-December 2008 (Table 1). The overall Māori:non-Māori rate ratio was 0.864 (95% CI 0.862–0.867). If Māori children had the same outpatient consultation rate as non-Māori there would have been 23,373 more consultations by Māori children annually. Māori children were nearly 30% less likely than non-Māori to attend outpatient mental health services. If Māori had the same consultation rate as non-Māori there would have been 5740 more consultations by Māori children per year in mental health services.

### Inequity in Accident Compensation Corporation (ACC) accident and injury consultations (2003–2007)

There were 291,502 Māori and 1,322,560 non-Māori ACC claims for children < 15years during the 2003–2007 period. Māori rates were significantly lower than non-Māori, with an overall rate ratio of 0.680 (95% CI 0.678–0.683). There were 26,442 fewer claims per year by Māori children in the period than if claims had been made at the non-Māori rate.

### Inequity in primary care (2007–2008)

A mean number of 353,734 Māori and 1,374,972 non-Māori children were enrolled in Primary Health Organisations (PHOs) during 2007–8; fewer in total than the SNZ population estimates for the same period, and a significantly lower proportion of Māori children than in official population estimates. If Māori children had consulted GPs at the same rate as non-Māori, there would have been 40,041 more GP consultations annually. Nursing consultations showed the inverse; although total nursing consultations were much fewer than GP consultations, Māori utilisation rates were 1.68 times greater than non-Māori, resulting in 17,194 consultations more than expected if Māori utilisation had been equivalent to the non-Māori rate.

### Inequity in pharmaceutical claims (2007–2008)

There were over 7.9 million pharmaceutical claims for children <15 years during 2007–8; only 21.7% of these were for Māori <15years. Māori rates of claims were significantly lower than non-Māori, representing an “under-utilisation” of 198,108 claims per year.

### Inequity in laboratory utilisation (2007–2008)

In 2007–8 there were 1.28 million laboratory claims for children <15years; 12.9% of these were recorded as being for Māori children. The overall Māori:non-Māori rate ratio was 0.454 (95% CI 0.452–0.455); rate ratios in children <1years were even lower. There would have been 101,922 more claims by Māori children each year if Māori had the same rate of laboratory use as non-Māori.

### Inequity in avoidable mortality

In the period 2003–7 there were 299,421 live births and 2,345 deaths in children aged 0- < 15yrs. 85% of these deaths were “potentially avoidable”. If Māori avoidable mortality rates had equalled non-Māori rates for each age group in the period, there would have been 333 fewer Māori deaths (nearly 67 deaths per year); 56% of these would be in the 28 day- <1year age group (Table 2).

**Table 2:** Avoidable Deaths by Ethnicity and Age Group, 2003–7

**Age group**	**Māori avoidable deaths (N)**	**Māori rate/100,000**	**Non-Māori avoidable deaths (N)**	**Non-Māori rate/100,000**	**Rate ratio (95% CI)**	**Expected Māori deaths if Māori rate equaled non- Māori rate**	**“Excess” Māori deaths (2003–7)**
**0- < 28days**	260	300.65	593	278.48	1.08 (0.93–1.25)	240.8	**19.17**
**28days- <1 year**	302	349.22	279	131.02	2.67 (2.27–3.14)	113.3	**188.69**
**1 year- < 5years**	98	33.62	139	16.43	2.05 (1.58–2.65)	47.9	**50.09**
**5years- < 15yrs**	132	18.53	184	7.98	2.32 (1.86–2.90)	56.8	**75.17**
**Total**	792	72.62	1195	35.50	2.05 (1.87–2.24)	n.a.	**333.12**

Māori avoidable mortality rates were significantly higher than non-Māori in all age groups except for the first month of life. The biggest contributor to mortality in this age group was deaths related to perinatal conditions (mainly resulting from prematurity), where Māori infant mortality rates were non-significantly higher than non-Māori.

In the age group 28days-<1year, 41.5% of the avoidable deaths were assigned “Sudden Unexpected Death in Infancy” (SUDI) codes. SUDI rates were significantly higher for Māori and especially Māori males in this age group; respiratory diseases (J00-99 codes) were the other main contributor to Māori infant deaths under one year.

There were significantly higher rates in Māori for “external causes” (accident, injury and assault codes V01-Y98) in all age groups, with the highest rates and rate ratios seen in infants 28 days - <1year. In girls 1–5years, the Māori:non-Māori rate ratio for injury/external causes was 3.35 (95% CI 2.00–5.62), and this accounted for nearly all the excess deaths. In 5–15year-old girls, nearly two thirds of the “excess” mortality was injury-related. In 5–15year-old males, injury/external causes again contributed to the majority of “excess” deaths, although the highest Māori:non-Māori rate ratios were for the ICD-10AM “A–B” and “E” codes (infectious and parasitic diseases, and endocrine, nutritional and metabolic diseases).

### Estimation of years of life lost from premature mortality

The years of life lost (YLL) to excess avoidable mortality in each age/sex group was computed, based on the difference between the midpoint of age of death in each age group and the non-Māori life expectancy for their same sex counterpart. The total value was 5210 life years lost per year. We examined the data using a range of discount values, based on those used in recent NZ cost of illness studies [33-37,42-44]. Applying a discount rate of 3% reduces the life years lost to 38% of their present value; if it is increased to 8%, it is only 16% of present value (831 life years) (Table 3).

**Table 3:** Years of Life Lost (YLL) from Avoidable Māori Child Deaths by Age Group and Gender, at Varying Discount Rates

**Years of Life Lost (YLL)**	**0%**	**3%“Base Case”**	**5%**	**8%**
0–1yr female	7279	2680	1726	1095
1–5yr female	1825	689	446	283
5–15yr female	2926	1182	776	497
**Total Female**	**12030**	**4551**	**2948**	**1875**
0–1yr male	9480	3626	2353	1498
1–5yr male	2091	820	535	341
5–15yr male	2450	1029	683	439
**Total Male**	**14021**	**5475**	**3571**	**2278**
**Total 2003–7**	**26051**	**10026**	**6518**	**4153**
**Annual YLL**	**5210**	**2005**	**1304**	**831**
Equivalent “Statistical Lives”*	**66.8**	**25.7**	**16.7**	**10.6**

*Assumes life expectancy of 78 years.

### Cost of years of life lost

The Method 1 “base case” scenario assumes a mean life expectancy at birth of 78 years, YLL discounted at 3%, and uses the New Zealand Ministry of Transport VoSL at June 2008 prices ($3,352,000) [36,42]. The YLL at 3% discounting are equivalent to the value of 25.7 “Statistical Lives” annually, computing to a cost of $NZ86.18 million annually at present value.

Method 2 results in similar values to Method 1 at zero discounting ($NZ223.3million per year), but as the VoSLY increases in Method 2 with increases in the discount rate, at 3% this computes to $NZ224 million per annum.

### Costs to society and family

The cost of work days lost by caregivers is based on the median length of stay for avoidable hospital admissions. This computes to $269 for each of the 3075 annual “excess” avoidable admissions, a total cost of $827,175 per year. Māori caregivers “out of pocket” payments for primary care were estimated to be $245,523 less than expected, primarily due to lower GP consultation rates for tamariki Māori.

Table 4 shows the total annualised cost estimate for each method.

**Table 4:** Comparison of Annualised Costs of the “Base Case” Scenario at 3% Discount Rate using Alternative Methods

**Cost category**	**Annual value ($NZ)**
Health sector costs	−$24,737,408
Loss of wages	$827,175
Out of Pocket Costs (Primary Care)	−$245,523
**Sub-total**	−$24,155,756
Method 1: Years of Life Lost	$86,181,425
Method 2 Years of Life Lost	$224,035,436
**TOTAL COST**	
**Method 1**	**$62,025,669**
**Method 2**	**$199,879,680**

### Sensitivity analysis

We examined the impact of different discount rates, using both methods. Method 1 results in a rapid decrease in present value as the discount rate increases. However using O’Dea & Tucker’s method there is little variability in the total value at differing discount rates (Table 5).

**Table 5:** Cost of Years of Life Lost (YLL) by Age group and Gender at Varying Discount Rates (Method 2) ($NZ)

**Cost of YLL**	**0%**	**“Base case” 3%**	**5%**	**8%**
0–1yr female	$294,130,013	$294,947,777	$294,391,755	$294,180,462
1–5yr female	$76,163,022	$76,394,291	$76,240,799	$76,179,257
5–15yr female	$133,695,626	$134,198,138	$133,878,164	$133,730,682
0–1yr male	$402,528,431	$403,786,759	$402,944,243	$402,597,057
1–5yr male	$91,790,026	$92,102,235	$91,897,812	$91,809,104
5–15yr male	$118,240,841	$118,747,980	$118,437,538	$118,282,829
**Total**	**$1,116,547,960**	**$1,120,177,180**	**$1,117,790,309**	**$1,116,779,391**
**Annual**	**$223,309,592**	**$224,035,436**	**$223,558,062**	**$223,355,878**

We also varied the “value of a statistical life year” (using values derived from previous studies [34-37]); predictably this has a significant impact on the final result, as the cost of “excess” avoidable mortality is a large proportion of the total costs (Table 6). The final range of estimates shows the total cost is highly sensitive to the method used, and the discount rate and VoSL applied.

**Table 6:** Value of YLL from Childhood Inequities, varying VoSL Values and Discount Rates ($NZ)

VoSL value (June 2008 prices)	0%		“Base case” 3%		5%		8%	
	Method 1	Method 2	Method 1	Method 2	Method 1	Method 2	Method 1	Method 2
$2.212m (Fire Safety, 2007 [43])	$147,797,553	$147,384,352	$56,879,748	$147,863,409	$36,981,626	$147,548,342	$23,563,447	$147,414,901
$3.352m (Transport 1991 [42])	$223,935,654	$223,309,592	$86,181,425	$224,035,436	$56,032,759	$223,558,062	$35,702,187	$223,355,878
$5.676m (Transport 1998 [36])	$379,192,367	$378,132,251	$145,931,824	$379,361,329	$94,880,802	$378,552,987	$60,454,853	$378,210,627

## Discussion

There have been many calls to reduce inequities in the health of New Zealand children [3,45-47]. In addition to social justice and ethical rationale for health equity, the economic costs that we bear through continued health inequities are important to consider. Assigning a monetary value to life or health remains antithetical to some. However, economic evaluation is commonly accepted as a consideration in decision-making, for example in allocation of government spending, and we believe this scoping study is an important initial step in developing more appropriate methods for examining the true costs of inequity.

While a preliminary attempt is described here, the costings that result can be considered highly conservative and an under-estimation of the full costs of inequity. Firstly, we have not assumed that all “avoidable” deaths and hospitalisations can be eliminated, but used a conservative counterfactual, estimating the number of potentially avoidable deaths/admissions/consultations that would have occurred if Māori children had the same rate as non-Māori in each age group.

Secondly, if non-Māori, non-Pacific children (i.e. predominantly New Zealand European) are used as the comparator group, greater inequities become apparent, given the high rates of illness and mortality experienced by Pacific children in New Zealand. 99 fewer deaths per year (nearly 77 of them Māori) and just under 10,000 hospital admissions would be prevented if Māori and Pacific children had the same rates of death and illness as non-Māori, non-Pacific children [data not shown].

In addition, we have not attempted to cost many of the childhood “diseases of inequity” such as rheumatic fever and bronchiectasis, which have lifelong impacts. We acknowledge that many of the significant social and intangible costs to children and families are not captured, including grief and suffering, missed educational opportunities, and employment and productivity losses for family, caregivers and for the child into the future.

The key findings, however, are important. Firstly, these estimates give an indication of the significant societal cost of inequities in health. As might be expected from similar economic analyses and other cost of illness studies, the human cost of the inequity in premature mortality is the greatest cost to society, rather than direct health system costs.

Secondly, health sector expenditure appears skewed towards non-Māori children. Our analysis suggests that it costs the health sector *less* to admit acutely sick Māori children, than to prevent severe illness through ensuring equitable primary care access or effective population-based interventions. Therefore a Ministry of Health concerned only with containing health sector spending has no incentive to reduce inequities in primary care access.

Lower utilisation of primary care and higher rates of potentially avoidable hospitalisations for Māori children are not new findings, despite persisting evidence of unmet need for primary care-amenable conditions [3,24,47]. Although primary care utilisation for all ethnic groups has increased since the introduction of the NZ Primary Health Care Strategy (2001) with additional funding to PHOs to improve financial access, the largest increase in utilisation has been by less deprived populations, where Māori are under-represented compared with non-Māori [48]. The reasons for poorer access to primary care are likely to be multi-factorial, including socio-economic factors. As primary care utilisation drives access to most other health services, including specialist outpatient services, addressing access barriers and attaining equitable utilisation of primary care services by Māori children has the potential to reduce the unacceptable disparities in avoidable hospitalisations and mortality seen here, and produce economic benefits that offset the costs of service delivery. Further intervention research in this area is crucial to understanding and addressing this inequity.

There are evident limitations in this study, and some unresolved challenges. Kaupapa Māori is a research methodology that utilises various research tools (in this case economic methods) to examine and contextualise Māori lived realities, to inform Māori development. Part of the spectrum of a Kaupapa Māori approach parallels Critical Theory and seeks to reveal inequity and challenge injustice. A major concern therefore in valuing child health and inequities relates to the values and assumptions of current economic approaches, and the appropriateness of the costing methods derived from these. The theoretical basis of “welfare economics”, which is the conventional neo-classical economic approach to social goods, is essentially utilitarian [27,36,49,50]. This assumes that the welfare output is maximised and is a function of individual preferences, where everyone is thought to maximise their own “utility” (i.e. the benefits they gain from their preferred choice of “goods”). This could be seen as antithetical to Māori values and concepts of reciprocity. It also presupposes that individuals are fully informed to make decisions in a free market, which is not an assumption easily applied to child health.

Further critique of this conventional welfare economics approach includes its indifference to the *distribution* of “utilities” (in this case, health states) across individuals and thus to concepts of equity, including intergenerational equity, which is important to consider in valuing child health [26,28,30]. Despite these limitations, it remains the conceptual basis for economic evaluation in the health sector.

Cost of illness methodology is descriptive, valuing in dollar terms the costs of a particular health problem, which then enables the economic burden of the problem to be estimated. Cost of illness studies are not considered full economic evaluations because they do not assess cost effectiveness or the cost-benefits of comparable interventions, and are critiqued by many welfare economists as not being sufficiently grounded in welfare economics theory. Other critique relates to the use of the human capital approach to evaluate the value of life. Despite these limitations, they can call attention to the importance of specific health issues, as demonstrated here [37,51].

A further area of debate in economic studies is how to value the loss of a life, as well as non-fatal outcomes such as the loss of function and lifetime sequelae of illness, especially for children [24,32,52-54]. There is some evidence that people may value a young person’s life more than an older person’s one [54,55]. Assigning a monetary value to life and health remains controversial, and there is considerable variation in “value of life” values obtained in empirical “willingness-to-pay” studies. We have used what is now regarded as a very conservative VoSL figure [35,56].

Discounting is another particular challenge in valuing child health. Discounting implies we value something more if we have utility from it today than in the future. For example, preventing the death of one infant achieves a gain of over 80 life-years, but this amounts to only 12 life years discounted at 8% (Treasury’s default rate in New Zealand [57,58]). There is considerable controversy about applying “market” discount rates to health, and ongoing debate about the assumptions underlying different discount rates [36,58]. Some argue that the discount rate should vary over time, rather than be applied at a constant rate, and it is not clear how sensible time preferences can really be for events over fifty years into the future [59]. It is unlikely that we would prefer to deny crucial preventive interventions for children, simply because the potential costs in terms of ill health would only be borne far into the future.

Data quality is another area of potential uncertainty. Although ethnicity coding in New Zealand has become more complete and accurate over the last decade, mis-classification and under-counting of Māori is still reported across the health sector [60]. In our data, there does not appear to be any net undercount of Māori in birth and mortality datasets [61]. Hospital numerator undercounting is estimated to be relatively small for young children, so we did not adjust for this [61]. For the laboratory, pharmaceutical, outpatient and ACC datasets there were small numbers of missing ethnicity values (<3%). In primary care, undercounting and misclassification of Māori persists [60,62,63], and is borne out in this study when enrolment data is compared with population estimates. Undercounting in the Census is described, especially of Māori and youth [64]; we used the Statistics New Zealand population estimates that are based on adjusted Census data, using post-census enumerator surveys to estimate the extent of undercount. Overall, ethnicity misclassification and undercounting of Māori is unlikely to have significantly altered the overall findings.

## Conclusions

Our study shows that in addition to being preventable, unnecessary and a breach of child rights, inequities in child health result in significant costs to our society. Effective interventions to address health inequities are available, although further investment in understanding and evaluating these is urgently needed. Improved access to primary care [65-67], better housing [68,69], lowering child poverty rates [46,70] and the provision of quality early childhood education and childcare [71] have been shown to impact positively on both child health and longer-term health outcomes. As inequities in adult health are closely associated with inequities in childhood health [46,72], strategies to reduce inequities in child health should be given high priority given the longer term social and economic benefits to be gained.

## Competing interests

The authors declare they have no competing interests.

## Authors’ contributions

All authors contributed to the study conception and design. CM led analysis of study data and drafted the manuscript. All authors read, revised and approved the final manuscript.

## Pre-publication history

The pre-publication history for this paper can be accessed here:

http://www.biomedcentral.com/1471-2458/12/384/prepub
